# The Incidence and Prevalence of Common Variable Immunodeficiency Disease in Taiwan, A Population-Based Study

**DOI:** 10.1371/journal.pone.0140473

**Published:** 2015-10-13

**Authors:** Chih-Wei Tseng, Kuo-Lung Lai, Der-Yuan Chen, Ching-Heng Lin, Hsin-Hua Chen

**Affiliations:** 1 Division of Allergy, Immunology and Rheumatology, Department of Internal Medicine, Taichung Veterans General Hospital, Taichung, Taiwan; 2 School of Medicine, National Yang-Ming University, Taipei, Taiwan; 3 School of Medicine, Chung-Shan Medical University, Taichung, Taiwan; 4 Institute of Biomedical Science and Rong Hsing Research Center for Translational Medicine, Chung-Hsing University, Taichung, Taiwan; 5 Department of Medical Education, Taichung Veterans General Hospital, Taichung, Taiwan; 6 Department of Medical Research, Taichung Veterans General Hospital, Taichung, Taiwan; 7 Institute of Public Health and Community Medicine Research Center, National Yang-Ming University, Taiwan; 8 Institute of Hospital and Health Care Administration, National Yang-Ming University, Taipei, Taiwan; University of Thessaly, Faculty of Medicine, GREECE

## Abstract

Common variable immunodeficiency (CVID) is one of the primary immunodeficiency diseases that occur in both children and adults. We present here a nationwide, population-based epidemiological study of CVID across all ages in Taiwan during 2002–2011. Using the International Classification of Diseases, Ninth Revision code 279.06, cases of CVID were identified from Taiwan's National Health Insurance Research Database from January 2002 to December 2011. Age- and sex-specific incidence and prevalence rates were calculated. A total of 47 new cases of CVID during 2002–2011 were identified. Total prevalence rose from 0.13 per 100,000 in 2002 to 0.28 per 100,000 in 2011. The annual incidence rate during 2002–2011 was 0.019 per 100,000. Cases were equally distributed between males and females and males mostly occurred in younger patients. This nationwide population-based study showed that the incidence and prevalence of CVID in Taiwan were lower than that in Western countries.

## Introduction

Primary immunodeficiency diseases (PIDD) occur primarily in infants and children and are characterized by recurrent infections. Previous reports showed that the incidence of PIDD in Taiwan is estimated to be 2.17 per 100,000 live births, which is lower than in Western countries, and antibody deficiencies were shown to be the most common cause of PIDD in Taiwan [1, 2]. Common variable immunodeficiency (CVID) is a PIDD related to defective immunoglobulin production. CVID affects both children and adults. Manifestations of CVID include recurrent infections [3], autoimmunity [4], and a variety of inflammatory disorders including granulomatous diseases [5], and malignancies [6, 7]. It was worth to note that autoimmunity may precede the diagnosis of CVID [8].

Multiple organs can be involved in CVID, and patients may have chronic lung disease, gastrointestinal, and liver disorders [7].

Intravenous or subcutaneous immunoglobulin (Ig) replacement, which is expensive and reimbursed by Taiwan's National Health Insurance, is currently considered the most effective treatment for preventing infections [9].

A number of epidemiological studies of CVID have been conducted in Europe using data from registries of primary immunodeficiency involving multiple medical centers. These studies analyzed online-based information and data were entered mostly by research assistants from different centers [10].

In Iran [11], epidemiological studies were based on data from a registry maintained at a single pediatric immunodeficiency center. In Japan [12] and Korea [13], nationwide data were collected via questionnaires from centers across the country. However, to our knowledge, nationwide population-based epidemiologic studies of CVID using claims data have not been previously reported.

In Taiwan, the National Health Insurance Research Database (NHIRD) is a convenient source of claims data suitable for population-based epidemiologic studies. We had previously reported population-based epidemiologic data on the risk of rheumatoid arthritis using the National Health Insurance Research Database (NHIRD)[14]. In the present study, we used the NHIRD to conduct a nationwide population-based epidemiological study of CVID, including mortality rates, across all age groups in Taiwan during 2002–2011.

## Materials and Methods

### Study design

This study employed a retrospective, cohort study design using claims data.

### Data source

On March 1, 1995, the Taiwan government implemented a mandatory National Health Insurance (NHI) program, which provides health care for over 98% of Taiwan's population of 23 million people [15]. The NHIRD consists of comprehensive NHI-related longitudinal claims data of all beneficiaries, including information on individual characteristics, admissions, ambulatory visits, prescriptions, diagnoses, and examinations. However, certain lifestyle data, such as tobacco and alcohol use, are not maintained in the database. The Bureau of NHI (BNHI) also established a registry system for patients with catastrophic or major illnesses, including cancers, autoimmune diseases, and immunodeficiency. Patients were enrolled in the catastrophic illness registry only after validation by specialists who reviewed their medical records, such as laboratory data including genetic tests, imaging findings, and pathology reports. This study utilized the ambulatory, inpatient, and enrollment files of patients who had a catastrophic illness certificate for CVID during 2002–2011 as the data source. Patient information was anonymized and de-identified prior to analysis. Thus, informed consent was not obtained. The Ethics Committee for Clinical Research at Taichung Veterans General Hospital approved this study.

### Study samples

We identified all newly diagnosed CVID patients (ICD9-CM 279.06) with a catastrophic illness certificate from January 1, 2002 to December 31, 2011 in Taiwan as the study samples.

### Statistical analysis

Age- and gender-specific incidence, prevalence, and death rates during 2002–2011 were calculated. Statistical analyses were conducted using SAS statistical software, version 9.2 (SAS Institute, Cary, NC).

## Results

As shown in Table 1, there were 47 new cases of CVID during 2002–2011, which included 24 females and 23 males. The new annual cases ranged from 1 to 5 for women and 0 to 4 for men. The incidence rate ranged from 0.01 to 0.04 per 100,000/year for ladies/women and 0.00 to 0.03 for boys/men.

**Table 1:** Incidence and prevalence of common variable immunodeficiency in Taiwan, 2002–2011.

	Total population (millions)			Incidence case			Prevalence case			Death case			Incidence (per 10^5^/year)			Prevalence (per 10^5^/year)		
Year	F	M	T	F	M	T	F	M	T	F	M	T	F	M	T	F	M	T
**2002**	11.38	11.62	23.01	1	2	3	9	20	29	1	0	1	0.01	0.02	0.01	0.08	0.17	0.13
**2003**	11.48	11.34	22.81	3	3	6	11	23	34	0	1	1	0.03	0.03	0.03	0.10	0.20	0.15
**2004**	11.38	11.18	22.56	2	0	2	13	22	35	0	0	0	0.02	0.00	0.01	0.11	0.20	0.16
**2005**	11.71	11.48	23.18	2	3	5	15	25	40	0	0	0	0.02	0.03	0.02	0.13	0.22	0.17
**2006**	11.79	11.54	23.33	2	3	5	17	28	45	0	0	0	0.02	0.03	0.02	0.14	0.24	0.19
**2007**	11.86	11.57	23.44	3	2	5	20	30	50	0	0	0	0.03	0.02	0.02	0.17	0.26	0.21
**2008**	11.93	11.62	23.55	3	0	3	23	30	53	0	3	3	0.03	0.00	0.01	0.19	0.26	0.23
**2009**	11.97	11.59	23.56	2	3	5	25	30	55	2	0	2	0.02	0.03	0.02	0.21	0.26	0.23
**2010**	12.01	11.60	23.61	1	4	5	24	34	58	0	0	0	0.01	0.03	0.02	0.20	0.29	0.25
**2011**	12.05	11.62	23.67	5	3	8	29	37	66	1	0	1	0.04	0.03	0.03	0.24	0.32	0.28

F, Female; M, Male; T, Total

Common variable immunodeficiency: ICD-9 code 279.06 with catastrophic illness certificate

**Fig 1:**
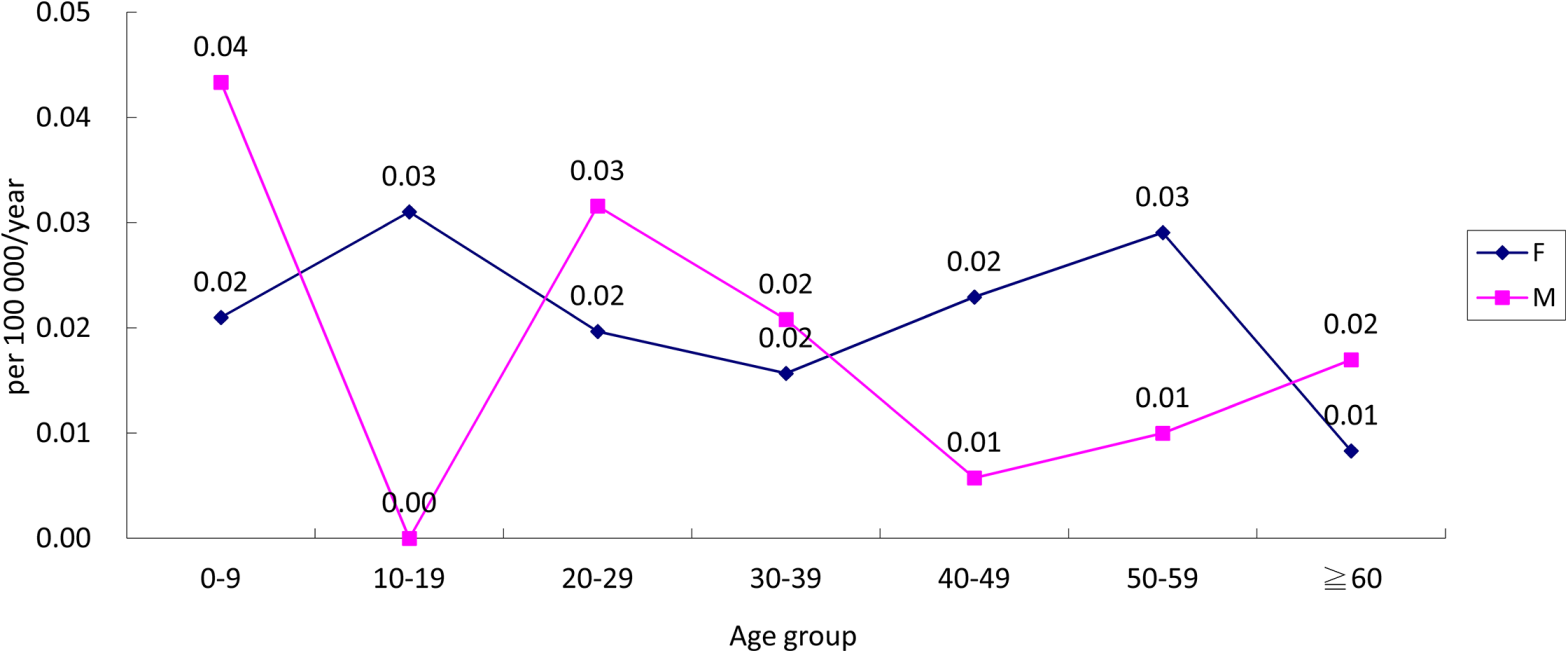
Age-specific incidence of common variable immunodeficiency in Taiwan during 2002–2011.

The annual incidence rate during 2002–2011 was 0.019 per 100,000. The age-specific incidence of CVID in Taiwan during 2002–2011 is listed in Table 2 and illustrated in Fig 1. There were nine new female cases and nine new male cases below the age of 20 years. For males, there were two incidence peaks: one in the 0–9 years age group and the other in the 20–29 years age group. Notably, no cases were observed in males in the 10–19 years age group. There were no significant peaks for females, but the incidence declined beyond age 60.

**Table 2:** Age-specific incidence of common variable immunodeficiency in Taiwan during 2002–2011.

	Case number			Person-years			Incidence rate (per 10^5^/year)		
Age group	F	M	T	F	M	T	F	M	T
**0–9**	4	9	13	19050769	20763786	39814555	0.02	0.04	0.03
**10–19**	5	0	5	16112058	17223667	33335725	0.03	0.00	0.01
**20–29**	4	6	10	20335978	19005695	39341673	0.02	0.03	0.03
**30–39**	3	4	7	19137192	19243247	38380439	0.02	0.02	0.02
**40–49**	4	1	5	17444663	17434274	34878937	0.02	0.01	0.01
**50–59**	3	1	4	10321238	10007054	20328292	0.03	0.01	0.02
**≥60**	1	2	3	12027651	11781940	23809591	0.01	0.02	0.01

F, Female; M, Male; T, Total

The total prevalence of CVID increased from 2002 to 2008 and from 2009 to 2011. The number of cases rose from 9 in 2002 to 29 cases in 2011 for females, and from 20 in 2002 to 37 cases in 2011 for males. The prevalence rate increased from 0.08 to 0.24 per 100,000, and from 0.17 to 0.32 per 100,000 for females and males, respectively. The total prevalence rate rose from 0.13 per 100,000 in 2002 to 0.28 per 100,000 in 2011.

Annual death cases are also listed in Table 1. Four men and four women died during 2002–2011. The annual mortality rate of CVID ranged from 0 to 0.03 per 100,000.

## Discussion

This study is the first nationwide population-based epidemiological investigation of CVID. The results showed that new cases (total 47) of CVID during 2002–2011 were equally distributed between males and females and mostly occurred at younger ages, especially in men. For females, the incidence was low beyond the age of 60. The overall prevalence of CVID steadily increased with time to 0.28 per 100,000 in 2011.

Our results are consistent with previous studies that showed cases of CVID were equally distributed in both sexes and occurred primarily in children. Its prevalence is estimated around 1:50,000 to 1:25,000 [16, 17]. Some registries of primary immunodeficiency disease have been established in Europe. A study of 2212 CVID patients using data from the ESID registry showed that both males and females had an approximately equal disease distribution, but when stratified into different age groups, there were approximately twice as many boys than girls [7]. However, male predominance with disease onset before age of 10 years in the registry raises the possibility that the patients may have undetected X-linked agammaglobulinemia with mutations in Bruton tyrosine kinase (Btk), and indeed some patients from certain centers had not been tested for Btk. Despite the possible presence of undetected X-linked agammaglobulinemia, it is possible that another X-linked defect underlies some patients with CVID but further studies are needed.

In Taiwan, patients with PIDD are often referred to medical centers where diagnostic evaluations can be completed, including tests for Btk. The diagnoses of CVID can be made if the followings are fulfilled: decreased srum level of IgG (at least two standard below the mean), and marked decrease in IgA, with or without low IgM, inability to produce protective level of specific antibodies, especially IgG, secondary causes of hypogammaglobulinemia have been excluded such as malignancies and exclusion of other primary immunodeficiencies. Immunodeficiency onset age at least two years of age and better more than four years. Patients (or their families) can submit related information to apply for catastrophic illness certificates. The application will be formally reviewed and valid for only five years. After validity period of 5 years, patients (or their families) have to reapply, and the application will be reviewed again to avoid misdiagnosis and misclassification.

According to Table 2 there are 13 chidren (4 females and nine males) were 0–9 years old. Among them one female and seven males were younger than four years at the time of application for catastrophic illness certificates. Diagnosis of CVID is made certain only after four years of age which is in line with the revision criteria by ESID. Onset age less than 4 years might include patients with transient hypogammaglobulinemia. But the catastrophic illness certificate validity lasts for only 5 years and after then patients (or families) have to send relevant information to reapply which diminished the possibility of transient hypogammaglobulinemia.

Two relevant studies have been conducted, and the results are consistent with our findings that the diagnosis of CVID was made at a younger age, and most cases were boys. One retrospective study on primary antibody deficiency in 34 patients at the Pediatrics Department of National Taiwan University Hospital with a follow-up of 20 years found that CVID and X-linked agammaglobulinemia were the most common disorders in patients with primary antibody deficiency [18]. The patients' diagnoses were evaluated by immunologic and molecular analysis. Btk genes were analyzed by polymerase chain reaction sequencing. There were 10 CVID patients, and 8 were male. Age at onset was 36.6 months + 21.76, and male predominance was noted. Our results showed consistent findings that boys were more likely to have CVID than girls in early childhood though CVID affected both sexes with respect to overall prevalence. Another cohort study was conducted in Chang Gung Memorial Hospital, which followed patients from 1985 to 2010 [19]. There were ten females and 16 males registered for CVID in the study in which disease onset was below age 15 in the majority of patients.

The prevalence of CVID in Taiwan increased steadily from 0.13 per 100,000 in 2002 to 0.28 per 100,000 in 2011, but the prevalence rate was still less than that in most Western countries and Iran. The ESID database showed that Germany had a prevalence rate of 0.5–0.6 per 100,000 inhabitants, France had 0.9–1.0 per 100,000, and United Kingdom had1.3 per 100,000 [10, 20–22]. In Iran [23], it was estimated that 1 in 91,000 persons had CVID. Though differences may exist due to different ethnicities and environmental factors, there is still underestimation of the disease burden. Patients with CVID might be concealed in the diagnosis of recurrent infections in different organs/ systems due to lack of awareness among general practitioners especially when patients are adults with mild or no symptoms [1]. Due to convenient medical service in Taiwan, infants and children are easily brought to medical centers, as birth rate in Taiwan continues to dip. However patients with adult onset may present with autoimmunity, enteropathy, granulomatous diseases or malignancies, and lack of awareness in internal medicine department might also lead to underestimation of CVID, but further studies are needed. As no conspicuous changes of incidence and death cases were found in our study, we believe that the prevalence rate may continue to grow. Immunoglobulin replacement is an effective treatment and has been shown to decrease the mortality rate. Higher immunoglobulin doses and maintaining higher IgG trough levels have been shown to significantly reduce infections [9]. With reduced infection rate and increased survival, the number of patients with CVID may continue to increase.

As shown in our results, more male patients developed CVID in early childhood while CVID developed in the 40–49 years and 50–59 years age groups in females. Like the possibility of X-linked PIDDs being classified as CVID in younger male patients, there is data to suggest that CVID is associated with autoimmunity. There was a case of common variable immunodeficiency mimicking rheumatoid arthritis with Sjögren's syndrome in a 26-year-old woman in Taiwan in which IVIG replacement therapy was ultimately successful in curing recurrent bacterial infections, chronic polyarthritis, and improving the severity of sicca syndrome [24]. Studies showed that more than 25% of CVID patients with autoimmune manifestations had cytopenia [25]. Furthermore, there is at least a subset of CVID patients with autoimmune cytopenia and lymphadenopathy could have an immune dysregulation such as autoimmune lymphoproliferatve syndrome. Somatic mutations of key elements in the immunity could lead to immune dysregulations and PIDDs with presentations of autoimmunity [26, 27]. Female predominance in this 40–59 years age group is also prevalent for autoimmune disease. Mutual causes underlying autoimmunity and CVID might be present. And the assumption that the female predominance in adult CVID relating to autoimmunity needs further studies.

This retrospective, population- based cohort study using claims data from the catastrophic illness registry in NHIRD is valuable for its data source with a good approximation of all cases of CVID. But there were limitations in this study. Firstly, the age of onset was not available in the NHIRD and the age distribution in the study merely represents the age at which approval for the catastrophic illness certificate was obtained. Patients might have had delayed diagnosis that could not be determined in this study. Secondly, the certificate for catastrophic illness is valid for five years, after which the patient needs to reapply. Thus, elderly patients may neglect to reapply for the certificate and this could result in an underestimation of the prevalence in adults. Thirdly, this study utilizes only catastrophic illness registration data and those patients with ambulatory diagnoses not fulfilling the requirement for catastrophic illness certificates may also lead to underestimation.

In conclusion, CVID is a heterogenous disease and some patients with early onset might present with recurrent infections while others with later onset might present with autoimmunities, enteropathies, or malignancies. Here, we show the incidence, prevalence, and mortality rates of CVID from nationwide health insurance and registry data. Male predominance in early childhood and rare occurrence of female CVID beyond age 60 were noted. Further studies on the associations between CVID and autoimmunities, enteropathies, and malignancies might reveal trends that may be of value to clinicians and public health policymakers.
